# State Medical Association of Texas

**Published:** 1906-05

**Authors:** 


### STATE MEDICAL ASSOCIATION OF TEXAS.

The thirty-eighth annual meeting of the State Medical Association of Texas, held at Fort Worth, Texas, April 24th, 25th, and 26th, was a record-breaker in point of attendance. It was the largest gathering of Texas physicians ever held. The register shows 522 present, but there were many who failed to register, and there is no way of ascertaining how many were there, first and last; the number is variously estimated at from 600 to 800.

The meeting was a gratifying success. The papers read were, for the most part, of a high order of excellence. As they will appear sooner or later in the Association's journal, together with the discussions, I can not go into a specific mention of them. The entertainment provided by the very active committees was delightful and much enjoyed. The annual banquet was dispensed with; five small loaves and seven small fishes—nay, five hundred of each would not have fed that multitude. It was *cut out,* and it is likely that we have seen the last of that feature. We may record, therefore, "the passing of the banquet." The "Red Back" has contended for years against it, seeing that the expense falls upon the local profession where the meeting takes place. It is burdensome, —and—useless. In lieu of a banquet and the usual feast of reason and flow of—wine or beer, the Fort Worth doctors entertained the multitude, members, ladies and visitors, at an out-of-doors *spread,* under the boughs of grand old oaks on grassy lawn; an old-fashioned barbecue. Five hundred doctors and ladies sat down to a feast as unique as it was enjoyable. Tender barbecued lamb, snowy bread, dripped coffee, with cream, and the usual trimmin's, such as chow-chow, olives, etc., made one's mouth water and satisfied five hundred appetites, sharpened by a hair-raising whirl of seven miles on the Interurban. All was jollity. The feast was spread on long tables in a beautiful grove at Handley, on the shores of

Lake Erie, and—well, it suggests Lake Como, and we are tempted to quote Bulwer, but we are not in the poetic vein just now.

The Fort Worth ladies entertained the visiting ladies delightfully at a series of luncheons, drives, and the Elks gave a dance.

MUCH IMPORTANT BUSINESS was done by the House of Delegates; so much, in fact, that that body was in almost continuous session. The sections have grown to such proportions and the time allotted to the meeting having been curtailed to three days, it was deemed advisable to double up some of the sections. The section on Railway Surgery will be merged into that of Surgery; and Obstetrics and Gynecology will be combined, cutting loose "Diseases of Children" from the first mentioned and merged into the section on Practice of Medicine, etc.

THE PASSING OF THE ORATOR.—Dr. W. S. Carter, of Galveston, was elected orator for next meeting, after which the office is to be abolished. He will be the last orator. As the president's address, the annual oration and the usual ball, reception or dance all are crowded into one evening, it was thought best to cut it out.

I can not speak of the president's address, or the annual oration by Dr. M. L. Moody, as I did not hear either. They will doubtless appear in the journal of the Association.

THE JOURNAL OF THE ASSOCIATION, inaugurated last meeting at Houston, in lieu of the annual volume of transactions, has been a gratifying success, and the reports of the secretary and the treasurer show the Association to be in a healthy financial condition. Much credit is due Dr. Chase, the indefatigable secretary-editor, for this success, but he nearly worked himself to death and has lost much avoirdupois and *embonpoint*—that fascinating rotundity that so well matched his bewitching little blonde Van Dyke beard. But—he'll rally. The trustees voted him additional office help,—one man. They should have doubled his salary. He is the right man in the right place.

THE MIXED BOARD BILL AND THE "RED BACK'S" LITTLE HATCHET. — The proposed bill for a State Board of Medical Examiners, in place of the three existing examining boards, which provided that the Governor should appoint eleven doctors, prorating them among the so-called

"schools," including all the pathies, came to grief without even a hearing. It was published exclusively in the "Red Back" in advance of the meeting, and a large edition was in the hands of its readers weeks before. The members thus had opportunity to study and digest it (although it transpired that a large number—a majority—could not "swallow" it). It seemed to be a surprise to most of those present, and it developed that there was such strong opposition to it that the committee having it in charge (Legislation and Public Policy) saw the wisdom of suppressing it. That is, they struck out all reference to "schools" and mention of pathies, all objectionable features, thus emasculating it and changing its character altogether. They altered it so as to provide for appointment by the Governor — without recommendation from the Association or any one else—of *eleven physicians,* to be a State Board of Examiners; examinations to be on all branches except Materia Medica and Practice (an absurdity—but in the interest of "harmony"). Well, it will not pass, anyhow. This is a very different thing, this having the Governor to select the board at his discretion,—and he *may* appoint all *physicians,* or—he *may* appoint all pathies, or he may mix them. We can't help it, but no physician is compelled to sit on a mixed board; and should such a thing come to pass, I doubt if any self-respecting physician will consent to affiliate with an assortment of pathies, more or less off color.

I wish to remind the profession of something that occurred twenty-eight years ago—to give my readers a piece of ancient history—an epoch-making event, a record and a precedent. At the annual meeting in Galveston in 1877, the late Dr. Harrison, presiding, Dr. Ross, of Brenham, one of the founders and fathers of the Association, an eminent and venerable member of the profession, *was arraigned for trial for accepting the appointment by the Governor—on a district board with a homeopath.* Dr. Jno. Pope, of Marshall (now living), and later, president, saved the old gentleman the disgrace of expulsion, by a scratch. But it drove him from the body, and he died not many years later.

I say, having the Governor to appoint a mixed board on his own initiative is a very different thing from the State Medical Association *asking* to be affiliated with men claiming to practice medicine on an exclusive and absurd dogma — and whose "sectarian title makes them quacks"—when every member of our Association knows that they are not eligible to membership by reason thereof, in either county or State associations.

It is a source of pride and congratulation, therefore, to believe and claim that the publication of the proposed monstrosity in the "Red Back," — the fearless champion of legitimate medicine,— high ethical character and defender of the traditions of the "regular,"—the only school of medicine—opened the eyes of the members, and thus prevented the House of Delegates from making a serious mistake, a stultification, a backward and downward step to the eternal disgrace of the profession; for, I believe, otherwise it would have passed the House. In doing so, I have antagonized some well-meaning but overzealous and thoughtless persons, and I have Blanche Tray and Sweetheart snapping at my heels. *Facilis descensus Averni.* It is easy-going with the current, down stream; but it requires courage and strength to stem it. One is liable to be swamped. I am done. I recognize a downward tendency—a tendency to homologate with every pathy— and even our honored president, in his address, says the day is coming soon when there will be but one medical profession,—meaning, doubtless, that we will absorb, by "benevolent assimilation," the heterogeneous mass—osteos and all.

Now, as to our colored brethren: will *he* be absorbed and become part of that one medical profession—as he is now part of the Great National? In Texas he is not eligible, under our by-laws, either to State or county medical societies, and can never be affiliated with the medical profession of Texas. But in the leveling process now going on,—dictated, doubtless, from Chicago,—and speaking by the mouth of the prophet of the Octopus, what are you going to do with him?

The Southern people — the medical men of the South — cheerfully accord to him all that is coming to him. We grant him all civil, political and religious rights, but the line is drawn and will never relax,—at social and scientific affiliation with him. The time has come for the Southern States to secede from the American Medical Association and establish a Southern Medical Confederacy! Who will start the organization of a Southern Medical Association?

Fees for Life Insurance Examination.—Many of the county societies having passed resolutions favoring a strike for a $5 minimum fee all over the State, it is a little disappointing that the House weakened and did not support them. A resolution to that effect was voted down and one agreeing to ask "reasonable compensation" for such service and for service to railroads, was adopted. It seemed to me to sound about like this: We ought to demand and receive $5; we think it is worth it. We don't want to do it for $3, the price offered by the insurance men, but if *we* don't do it, the homeopaths *will*, and—well, rather than that—we'll just do it. I quote from the Fort Worth newspaper the following:

Quite a lively discussion took place upon the question of the reduction of the examination fees by the insurance companies all over the country, which resulted in a resolution introduced by Dr. D. R. Fly, of Amarillo, being unanimously adopted, which resolution, as stated by Dr. Fly, who made a speech in which he created much laughter by his reference to a bobtailed Panhandle bull in fly time, recommends that the House of Delegates indorse and encourage the action of all State physicians in their efforts to secure reasonable compensation from railroad companies, insurance companies and fraternal orders for physical examinations.

In presenting this resolution for the consideration of the House, Dr. Fly said: "Gentlemen, what we need in this State is a remedy which we have in the Panhandle country, called 'stiffine.'

"When a physician is properly inoculated with this famous 'stiffine' it has an effect of stiffening the spinal column of the patient until he has backbone enough to stand up and ask for his dues. If any member of this Association has no other means of securing this wonderful medicine, we of the Panhandle country can supply the entire State of Texas with enough to inoculate every member of this Association."

FLY'S STIFFINE.—The Association, it seems to me, does need this treatment, not only in this matter, but in the matter of asking for legislation. By the bye, it will be in order for Dr. Fly to publish the formula of Stiffine or he will get the Octopus fellows after him.

A BIG SOUTHWESTERN MEDICAL ASSOCIATION PROPOSED.—Dr. Jabez Jackson, of Kansas City, ex-president of the Missouri State Medical Association, by invitation delivered an address to the House of Delegates. Dr. Jackson highly complimented the Texas doctors and said he would have to admit that Texas did better in the way of good association work than did many of her sister States. He proposed the organization of a Southwestern Medical Association, claiming that the National organization had become so large that it is unwieldy and dominated by Eastern talent. His address was heartily applauded and at the close of his remarks, on a motion duly made and seconded, the chairman appointed a committee of five members to confer with similar committees which will be appointed by the State medical organizations from Missouri, Kansas, Indian Territory, Oklahoma Territory, and Arkansas.

Dr. Jackson is a son of the late Dr. J. T. Jackson, of Sedalia, Mo., once chief surgeon of the Missouri Pacific system of railroads, well remembered by the older members as a sterling man and a typical Southern physician. Dr. Jabez is a chip off of the old block, and we heartily endorse his effort and want to see it succeed, and then see this new society drop off from the A. M. A., which it will surely do. Dr. Jackson said: "We are tired sitting at the feet of the Eastern men, and being made to feel that the East is the sole source of wisdom. The West nominated Mayo, and we were going to have him, or bust the thing up," or words to that effect.

THE ASSOCIATION OUT IN THE COLD.—No city seemed to want the next meeting; it is too big a proposition, now that it has grown to such proportions. Not an invitation was presented except an *ex parte* one from the members from Mineral Wells. It was enthusiastically accepted. It was Hobson's choice. We have never met there, and it remains to be seen how the Wells people are going to handle the crowd. It was Dallas' time, or Waco, but—nothin' doin'.

MINERAL WELLS, next place of meeting. Time, first Tuesday, Wednesday, and Thursday in *May,* 1907.

OFFICERS ELECT.—President, Dr. G. B. Foscue, of Waco; Vice-Presidents, Dr. Felix Miller, of El Paso, Dr. B. S. Wier, of Beaumont, and Dr. A. B. Small, of Waxahachie. The Secretary and Treasurer hold over. The President has not yet announced the committees and section officers. New Councilors: S. T. Turner, J. M. McCarver, H. W. Cummings, J. A. McCracken. Delegates A. M. A.: Wilson, Cantrell, A. C. Scott, Paschal, Turner.

THE REPORTS OF THE FIFTEEN COUNCILORS show a satisfactory condition in most counties. In some there have been gains in membership, in some, losses, but altogether a net gain in the State in enrollment, of over 500. This brings the total membership up to 2850.

THIS IS VERY GRATIFYING. We have a splendid body and every one should exert himself to further build it up and keep it built up. The greatest menace to it is the liability of members, from lukewarmness or indifference, to fail to pay their annual dues, and be dropped. This would be fatal. The secretaries had much difficulty this year in rounding them up, and I fear the secretaries will tire of the job of having to run after stragglers to get the little $2, or—advance it for them—as I know was done in one case.

ANCHOR IT.—Now, let us locate. The Association, numbering nearly 3000, with 600 to 800 in attendance, is too "hefty" and unwieldy to box the compass, as we have been doing with a small membership these twenty or thirty years—swinging around Dallas, Fort Worth, Waco, Austin, San Antonio, Houston, and Galveston, and back again every seven years. The Association should have a home. It should have a library and a pathological museum and own its own building and printing outfit. It should meet every year at Austin, for instance, and pay its own expenses—and not expect the local physicians or citizens to entertain them. Besides, as said before, nobody wants the meeting at the price.

THE HOUSE OF DELEGATES enthusiastically endorsed the administration of State Health Officer Tabor, and gave him a rising vote of thanks for his efficient services last summer in keeping Texas free from yellow fever.

